# Comparison of physical preservation strategies for accurate characterization of the coffee fruit microbiome

**DOI:** 10.1007/s00203-026-05046-7

**Published:** 2026-07-13

**Authors:** Etztli Itzel Morales Reyes, Tomás Gomes Reis Veloso, José Maria Rodrigues da Luz, Monalisa Araujo Aziz, Martín Alejandro Bolaños González, Marliane de Cassia Soares da Silva

**Affiliations:** 1 Colegio de Postgraduados, Postgrado en Hidrociencias, Campus Montecillo. Carretera México-Texcoco, Montecillo, Texcoco, Estado de México; 2 Departamento de Microbiologia, Laboratory of Mycorrhizal Associations – LAMIC, Universidade Federal de Viçosa, Avenida PH Rolfs S/N, Viçosa, Viçosa, CEP, 36570-000 Minas Gerais Brazil; 3 LAMIC, DMB, UFV, Ph Rolfs Avenue S/N, Viçosa, 6570-000 Minas Gerais, MG Brazil

**Keywords:** DNA sequencing, Microbial diversity, *Coffee arabica*, Cryopreservation, Lyophilization

## Abstract

The method used to preserve samples prior to DNA extraction is crucial for the accurate characterization of microbial diversity. This study evaluated the effects of different storage conditions on microbial DNA preservation in *Coffea arabica* fruit samples. Coffee cherries were harvested directly from plants, placed in plastic tubes, and stored at 4 °C before being subjected to four treatments: lyophilization, cryopreservation at − 80 °C, and refrigeration at 4 °C. Samples were stored for 3 and 13 days. Microbial communities were characterized by next-generation sequencing. Lyophilization retained more than 80% of the ASVs shared between days 3 and 13 of storage, whereas refrigeration at 4 °C retained less than 50%. These findings demonstrate that preservation method significantly affects the integrity of bacterial and fungal microbiomes in *C. arabica* beans. The absence of a time-zero control, chemical preservative comparisons, and the study’s limited scope (single variety, location, and short storage period) warrant cautious interpretation of the findings. Although lyophilization was the best-performing physical preservation strategy evaluated here, broader validation across coffee cultivars, environments, and storage durations is still required before it can be considered a standard preservation protocol for preserving microbial materials derived from coffee samples.

## Introduction

The microbiome associated with coffee fruits plays a fundamental role in shaping the sensory profile of coffee beverages, as microbial communities strongly influenced by terroir directly affect fermentation processes and, consequently, the final quality of the product (Rojas-Chacón et al. 2025; Wiele et al. 2025). Deepening our understanding of this microbiome, both as an integrated system and at the level of its individual components, represents a strategic pathway for harnessing its biotechnological potential and contributing to the long-term sustainability of coffee production (Vaughan et al. 2015; Veloso et al. 2020). However, for coffee microbiome studies to be comparable and reproducible, it is essential to ensure sample integrity from the moment of collection through to molecular analysis.

In this regard, high-throughput sequencing (HTS) technologies have substantially expanded the capacity to characterize microbial communities in plants, soils, and agricultural systems. Nevertheless, their rapid adoption has led to considerable heterogeneity in protocols for sample transport, processing, and preservation — an aspect that has received relatively little attention, even though inadequate preservation can cause DNA degradation and substantially alter the apparent composition of bacterial and fungal communities. This challenge is particularly relevant in agricultural systems such as coffee, where sampling sites are often located in remote areas, far from the laboratories where DNA extraction takes place. Sample preservation is a critical component of microbiome studies, as pre-analytical conditions directly influence DNA quality and the composition of the microbial communities detected. With the development of next-generation sequencing (NGS), it has been recognized that biases introduced during collection, storage, and transport can significantly affect the reproducibility and comparability of results across studies (Fouhy et al. 2015).

Immediate processing of fresh samples is considered the gold standard in microbiome studies, as it minimizes post-sampling changes associated with differential bacterial growth and DNA degradation. However, in field studies and remote areas, immediate processing is rarely feasible; therefore, freezing at − 80 °C has become the most widely used method for microbial sample preservation (Lauber et al. 2010; Choo et al. 2017; Hale et al. 2015). While this method inhibits microbial metabolic activity and helps maintain DNA integrity, it may also introduce variations in microbial diversity depending on storage time and conditions.

Several comparative studies have demonstrated that variables such as temperature, oxygenation, and the use of chemical preservatives can modify alpha and beta diversity, as well as the relative abundance of taxa and detected metabolic functions. Jenkins et al. (2018) reported that different storage strategies generate specific post-collection biases affecting both taxonomic composition and inferred metabolic pathways. In line with these findings, Shaw et al. (2016) found that storage conditions can introduce greater variation than storage time or even the sequencing platform used, underscoring the need to standardize methodological protocols.

To facilitate preservation in field settings, commercial reagents such as DNA/RNA Shield™, RNAlater^®^, and OMNIgene·GUT has been developed, allowing nucleic acids to be stabilized at room temperature (25 °C) or under refrigeration. However, due to their cost, more affordable alternatives are also employed, including absolute ethanol, air-drying, and homemade preservation buffers; these options, nonetheless, may introduce additional biases into the structure of the microbial community (Camacho-Sanchez et al. 2013; Guo et al. 2016).

For studies involving long-distance transport, lyophilization has been proposed as a promising strategy, as it allows samples to be preserved at room temperature without the risk of thawing. This method removes water from the sample through sublimation under vacuum, halting microbial metabolic activity and reducing sample mass, theoretically allowing indefinite storage at room temperature (Nechvatal et al. 2008). Its effectiveness, however, is conditioned by the extraction method used and the size of the DNA fragments analyzed; therefore, its use in microbiome research must be accompanied by rigorous methodological controls to distinguish preservation-related biases from true biological variation.

Taken together, the available evidence indicates that no universal preservation method is free of biases, and that protocol selection must account for the study context, available resources, and the type of molecular analysis planned. Accordingly, the standardization and detailed documentation of preservation conditions are considered fundamental priorities in modern microbiome research, in order to ensure reproducibility and the correct biological interpretation of data (Fouhy et al. 2015; Jenkins et al. 2018). Within this framework, coffee fruit samples are collected directly in the field and transported refrigerated to the laboratory to minimize post-sampling changes. These samples are subjected to four preservation treatments: (1) freezing at − 80 °C of whole fruits; (2) lyophilization of macerated fruits; (3) refrigeration at 4 °C of whole fruits; and (4) lyophilization of whole fruits. The systematic comparison of these treatments will allow determination of which storage conditions most faithfully preserve the composition and diversity of coffee-associated microbial communities, contributing to the evaluation of physical preservation strategies for coffee fruit microbiome studies. Therefore, this study evaluates the impact of different storage conditions on the integrity of microbial DNA in coffee fruit samples, with the aim of providing comparative information on physical preservation methods applicable to field sampling.

## Materials and methods

This study was performed with coffee fruits (*Coffea arabica*, variety Catuaí Vermelho) produced on a coffee farm, in the municipality of Araponga, Minas Gerais state, Brazil (20° 45’ 48.1” S, 42° 33’ 59.5” W). This region is located at an altitude of 900 m above sea level and covers an area of 4,000 m² with a westward slope. The farm has been cultivating coffee for over 15 years using mineral fertilization management. In the 2023/2024 harvest, 1200 kg of NPK fertilizer was applied in the 25-5-20 ratio, divided into two equal doses of 600 kg (Entringer et al. 2026). Pruning is carried out every two years. Coffee beans were manually harvested in May 2024 (autumn), a period characterized by low rainfall (~ 50 mm), an average temperature of 25 °C, and relative humidity of 81%.

### Sample collection and processing

**Fig. 1:**
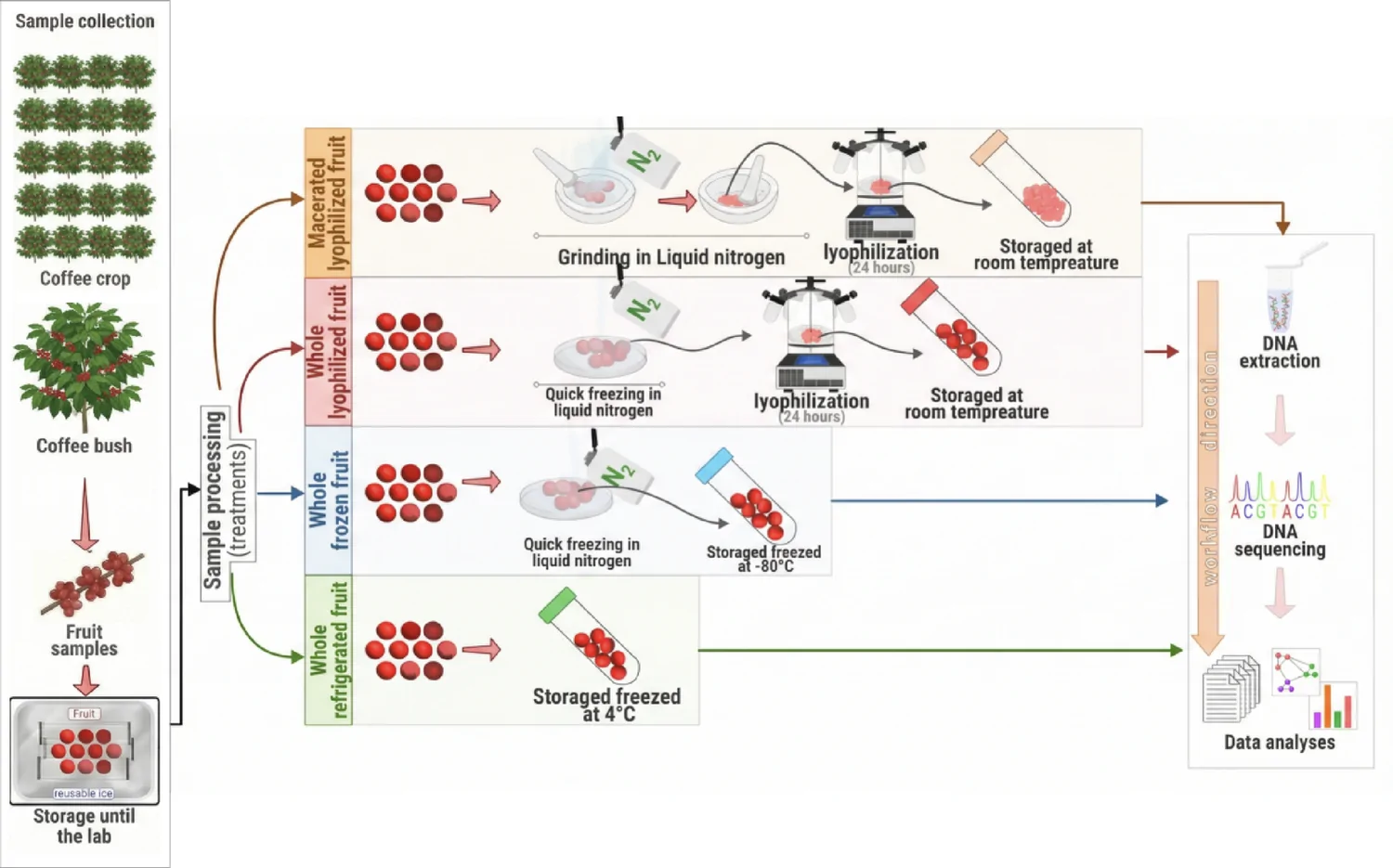
Experimental design to assess different treatments for coffee sample preservation. All samples had DNA extracted for microbial community analysis via 16 S and ITS sequencing after 3 and 13 days of storage

The coffee cherries were harvested directly from the coffee plants, placed in plastic tubes (50 ml Falcon tubes), and stored at 4 °C for transport to the laboratory (Fig. 1). This procedure minimizes biochemical and microbiological changes in the samples during transit. No fermentation process was applied to the coffee fruits.

The samples were distributed into four treatments (Fig. 1). For each treatment 4 true biological replication were performed, each one was composed of five coffee cherries in a plastic tube. The time elapsed before this processing was 4 h after the coffee fruits were collected.

In the treatment 1, coffee fruits were ground in liquid nitrogen, lyophilized for 24 h, and stored at 25 ± 3 °C. In the treatment 2, whole coffee fruits were frozen in liquid nitrogen, lyophilized for 24 h, and stored at 25 ± 3 °C. In the treatment 3, the whole coffee fruits were also rapidly frozen in liquid nitrogen and maintained at − 80 °C. In the treatment 4, the whole coffee fruit was stored at 4 °C without any prior processing. These treatments were stored for 3 and 13 days, in accordance with the study by Lauber et al. (2010). These samples were processed using the same analytical workflow: DNA extraction, sequencing using the 16 S rRNA and ITS markers for the characterization of bacterial and fungal communities, respectively, and bioinformatic analysis of the generated data.

### DNA extraction, amplification, and meta-amplified sequencing

Two hundred and fifty milligrams of fruit material (comprising four individual samples) underwent DNA extraction using the NucleoSpin Soil Kit (Macherey-Nagel, Germany). Prior to extraction, the fruit samples were frozen in liquid nitrogen. In the initial extraction step, the material was subjected to cell lysis using the Precellys^®^ 24 homogenizer (Bertin Instruments) at 4000 rpm for 50 s. Subsequent steps were follow the manufacturer’s instructions. After extraction, the integrity and quantity of the DNA was assessed via agarose gel electrophoresis (0.8%) stained with ethidium bromide.

To evaluate the microbial community, regions V3/V4 of the 16S rRNA gene and ITS1 were amplified through PCR using the primer pairs 341F (5’-CCTAYGGGRBGCASCAG-3’) / 806R (5’-GGACTACNNGGGTATCTAAT-3’) and ITS1F (5’-CTTGGTCATTTAGAGGAAGTAA-3’) / ITS2 (5’-GCTGCGTTCTTCATCGATGC-3’). The PCR products were analyzed by gel electrophoresis on 2% agarose gels stained with SYBR Green. Fragments corresponding to sizes between 400 and 450 base pairs (bp) were selected for library preparation for sequencing using the NEBNext^®^ UltraTM DNA Library Prep Kit, following the manufacturer’s protocol for the Illumina platform. Libraries were barcoded, and their quality were assessed using a Qubit 2.0 fluorometer (Thermo Scientific) and the Agilent Bioanalyzer 2100 system. These libraries were sequenced on the Illumina NovaSeq 6000 platform by Novogene (Hong Kong, China) with paired-end reads of 250 bp.

### Bioinformatic analysis

Sequencing reads was undergo adapter and barcode removal, followed by quality filtering to eliminate low-quality sequences, chimeras, and singletons (sequences that appear only once in a sample). High-quality sequences were processed using the Divisive Amplicon Denoising Algorithm (DADA2; Callahan et al. 2016) to identify Amplicon Sequence Variants (ASVs). Taxonomic assignments were performed using bacterial (SILVA 138; Quast et al. 2013) and fungal (UNITE; Nilsson et al., 2019) reference databases. Contaminant sequences originating from mitochondria, chloroplasts, or other non-fungal eukaryotes were removed. All subsequent analyses—including microbial diversity, relative abundance of taxa, and community structure—were performed in the R software (R Core Team 2020).

### Taxonomic classification and nomenclature

Taxonomic classification was performed according to the Genome Taxonomy Database (GTDB), and nomenclature follows the rules of the International Code of Nomenclature of Prokaryotes (ICNP), ensuring consistency across all taxonomic ranks.

### Experimental design and statistical analysis

The assay was performed in a completely randomized design with 4 treatments, 2 storage times, and 4 true biological replication. PERMANOVA was calculated with 1,000 permutations comparing the samples of the same treatment at different storage times at 0.05 level of significance. The higher the calculated F value, the greater the observed change in the bacterial community. The beta diversity index was obtained by Principal Coordinates Analysis (PCoA) based on the Bray-Curtis metric. Alpha diversity was assessed using richness (based on ASVs) and the Shannon diversity index. The difference statistical of the alpha diversity index was performed by Student’s t-test at 0.05% level of significance. The alpha and beta diversity were used to compare the diversity of microbial communities in the treatments.

## Results

The percent of sequences kept at each step of processing is shown in supplementary tables S1 and S2. Sequencing quality was sufficient to capture the microbial diversity present in the samples. It can be verified in these tables where the total raw reads sequenced, retained after filtering steps and the amount detected as chimeras are described. Sequencing data is deposited on the BioProject PRJNA1479068 (https://www.ncbi.nlm.nih.gov/bioproject/PRJNA1479068).

Lyophilization treatments retained more than 80% of the bacterial ASVs shared between days 3 and 13 of storage. When lyophilization is performed on macerated fruit, this percentage increases to nearly 90%. Conversely, preservation through refrigeration at 4 °C results in a loss of more than 50% of the bacterial ASVs shared between days 3 and 13. The F-values from PERMANOVA confirm this observation, showing the lowest F and, consequently, the least variation for the three lyophilized treatments.

### Microbial beta diversity

**Fig. 2:**
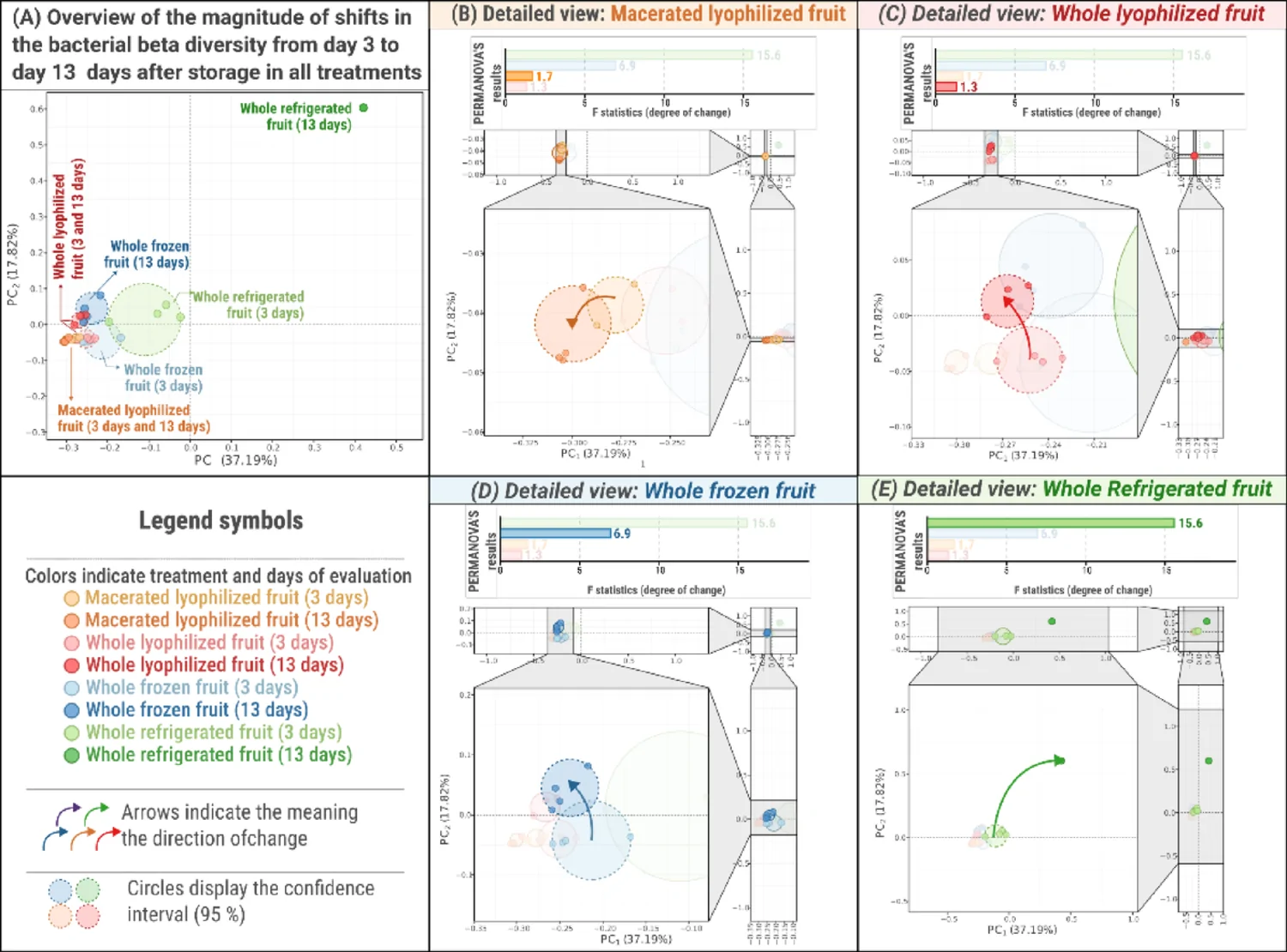
Beta diversity from bacterial community of coffee cherries subjected to different treatments for preserving microbial genetic material. Panel “A” shows the overall beta diversity profile across all samples, while the other panels (B, C, D, and E) detail the observed variation for each treatment over the two evaluated times. Circles represent the confidence interval and arrows indicate the direction of change in the microbiota (based on the centroid of the points)

The PCoA based on the Bray-Curtis metric revealed notable differences in the magnitude of change in bacterial beta diversity between days 3 and 13 of storage, depending on the preservation treatment applied (Fig. 2). Overall, the lyophilization treatments were closer to each other across both evaluation time points, while refrigeration at 4 °C exhibited the greatest shift in bacterial community composition (Fig. 2A).

When analyzing the treatments individually, the lyophilized macerated fruit recorded an F-value of 1.7, with partially overlapping confidence circles and a small displacement, confirming that both lyophilization treatments effectively preserved the structure of the microbial community over storage times (Fig. 2B). Similarly, the lyophilized whole fruit presented the lowest PERMANOVA F-value (F = 1.3), accompanied by broad overlap between the 95% confidence intervals for days 3 and 13, indicating that the bacterial composition remained practically stable throughout the evaluated storage period (Fig. 2C). However, the whole fruit frozen at − 80 °C showed an F-value of 6.9, with clearly separated confidence intervals between the two time points, reflecting a moderate change in beta diversity despite being the preservation method most commonly used as a reference in microbiome studies (Fig. 2D). The most pronounced change in the preservation of microbial genetic material was observed in the refrigeration treatment at 4 °C of whole fruits, which recorded the highest F-value (F = 15.6), with completely separated circles and the longest arrow, indicating a substantial alteration of the bacterial community between days 3 and 13 of storage (Fig. 2E). Therefore, lyophilization treatments of both whole and macerated fruits are the methods that best preserve the stability of bacterial beta diversity over time, while refrigeration at 4 °C proves to be the least suitable condition for maintaining the integrity of the microbial community in *C. arabica* fruit samples over time, as it fails to minimize variations in community structure.

**Fig. 3:**
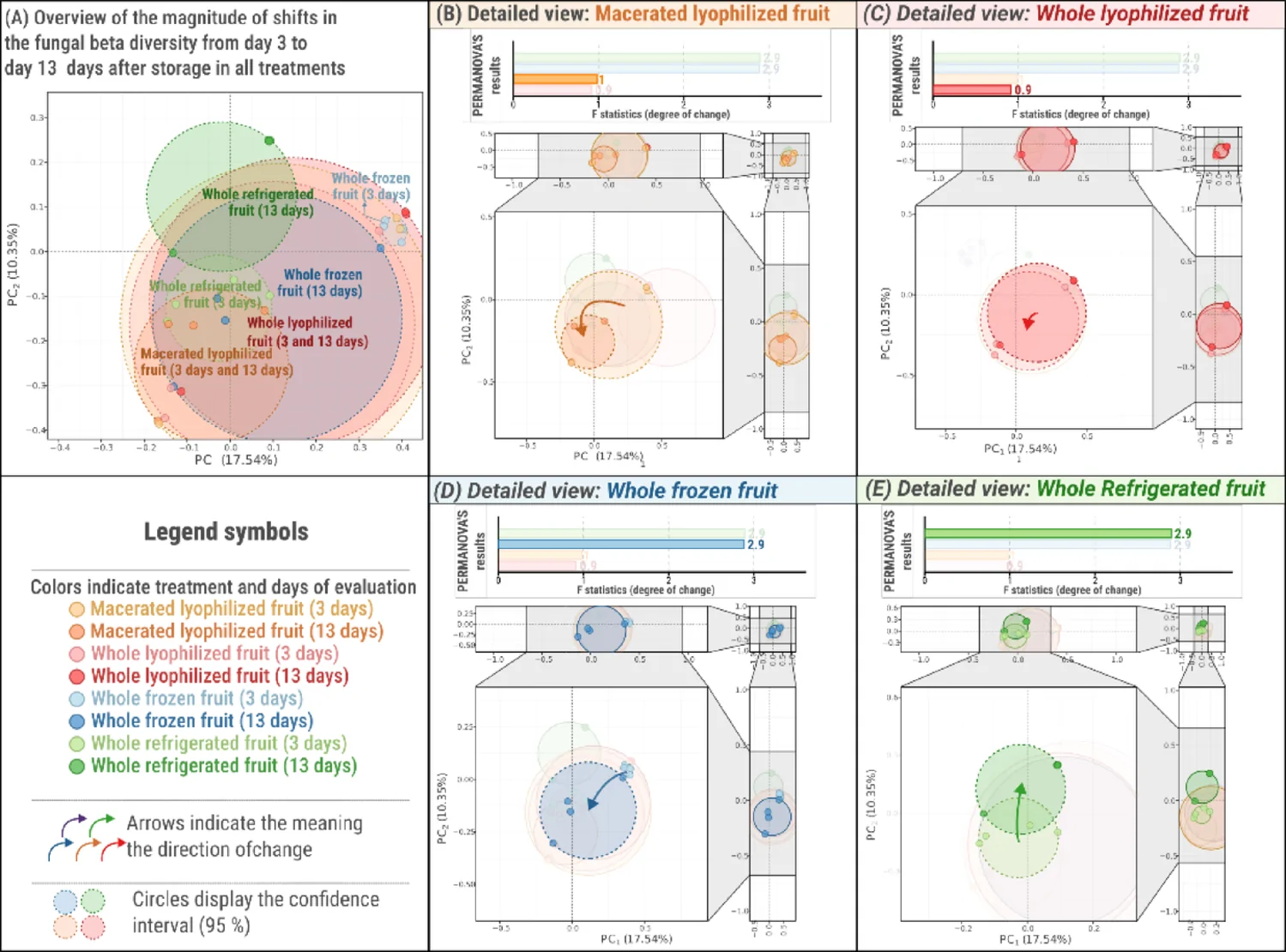
Beta diversity from the fungal community of coffee cherries subjected to different treatments for preserving microbial genetic material. Panel “A” shows the overall beta diversity profile across all samples, while the other panels (B, C, D, and E) detail the observed variation for each treatment over the two evaluated periods (3 and 13 days). Circles represent the confidence interval and arrows indicate the direction of change in the microbiota (based on the centroid of the points)

PCoA of fungal beta diversity revealed a general pattern of greater temporal stability compared to bacterial communities (Figs. 2 and 3). There was a significant difference in fungal diversity between the genetic material preservation treatments (Fig. 3). Lyophilization treatments — both whole and macerated fruits — were closely clustered between days 3 and 13, with notable overlap in their 95% confidence intervals, contrasting with the greater dispersion observed in the freezing and refrigeration treatments (Fig. 3A).

Lyophilized macerated fruit recorded an F-value of 2.0, with partially overlapping confidence intervals and a moderate displacement (Fig. 3B). Although this value is higher than that of the lyophilized whole fruit, the overlap between confidence circles suggests that the change in the fungal community between both periods is not statistically robust, indicating acceptable preservation, albeit slightly inferior to its non-macerated counterpart.

The lyophilized whole fruit presented the lowest PERMANOVA F-value among all fruit treatments (F = 0.9), with complete overlap of confidence intervals between both evaluation time points and a virtually null displacement (Fig. 3C). This result indicates that the fungal community composition did not undergo statistically detectable changes between days 3 and 13, positioning this treatment as the most effective for preserving mycobiome stability over time.

The whole fruit frozen at − 80 °C (Fig. 3D) and the whole fruit refrigerated at 4 °C (Fig. 3E) presented identical F-values (F = 2.9), the highest among the evaluated fruit treatments. In both cases, the confidence intervals showed clearer separation between days 3 and 13, and the displacement arrows exhibited greater length and a defined direction, indicating a more pronounced reorganization of the fungal community during storage. Notably, freezing at − 80 °C and refrigeration at 4 °C induced an equivalent magnitude of change in fungal beta diversity, suggesting that, unlike what was observed for bacterial communities, storage temperature did not exert a differential effect on mycobiome stability between these two treatments.

In summary, treatments ranked by their capacity to preserve fungal community stability place lyophilized whole fruit and lyophilized macerated fruit at the top (Fig. 3). These results are consistent with those obtained for bacterial communities (Fig. 2) and reinforce the conclusion that lyophilization — particularly of whole fruits — constitutes the most effective conservation strategy for maintaining the integrity of both the bacterial microbiome and the mycobiome in coffee fruit samples.

### Alpha diversity metrics

**Fig. 4:**
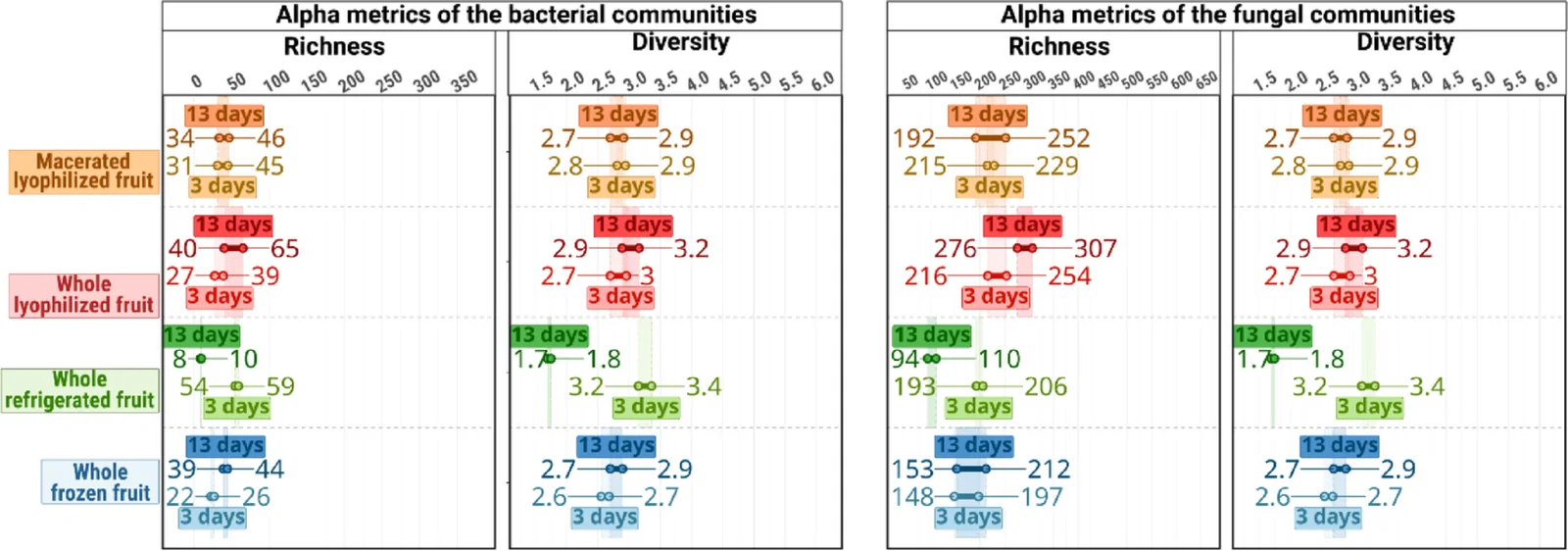
Alpha diversity metrics (richness and diversity) from the microbial community of coffee cherries subjected to different treatments for preserving microbial genetic material. The numbers on the left indicate the lowest values observed, while those on the right represent the highest values

Bacterial ASV richness was lower in the refrigerated whole fruits at 4 °C than in all other preservation treatments (Fig. 4). Furthermore, this treatment exhibited the lowest ASV richness values at day 13 compared with day 3 of storage (Fig. 4), indicating a greater reduction in detectable bacterial richness under refrigeration than under any other condition evaluated. These findings suggest that refrigerated storage progressively altered the bacterial community structure throughout the study period. In contrast, lyophilized whole fruits and whole fruits frozen at − 80 °C showed an increase in bacterial ASV richness between storage days, while lyophilized macerated fruits maintained comparable values at both time points (Fig. 4). Together, these patterns suggest that lyophilization and freezing confer greater temporal stability to bacterial richness relative to refrigeration.

Bacterial diversity, estimated using the Shannon index, followed a similar trend. Refrigerated whole fruits exhibited the most pronounced temporal change, with a marked decrease between day 3 and day 13 that reflected not only a reduction in ASV richness but also a decline in community evenness (Fig. 4). Lyophilization treatments, by contrast, yielded more stable Shannon values over time: lyophilized whole fruits ranged from 2.7 to 3.0 on day 3 to 2.9–3.2 on day 13, while lyophilized macerated fruits maintained nearly constant values across both storage times. Whole fruits frozen at − 80 °C showed a slight increase in Shannon diversity, from 2.6 to 2.7 on day 3 to 2.7–2.9 on day 13.

Fungal communities exhibited higher observed ASV richness than bacterial communities in all treatments (Fig. 4). Refrigerated whole fruits at 4 °C showed the greatest reduction in fungal ASV richness, declining from 193 to 206 ASVs on day 3 to 94–110 ASVs on day 13, a loss accompanied by a substantial decrease in the Shannon index from 3.2 to 3.4 to 1.7–1.8 — a pattern consistent with that observed for bacterial communities.

### Microbial taxonomic composition and shared ASVs

**Fig. 5:**
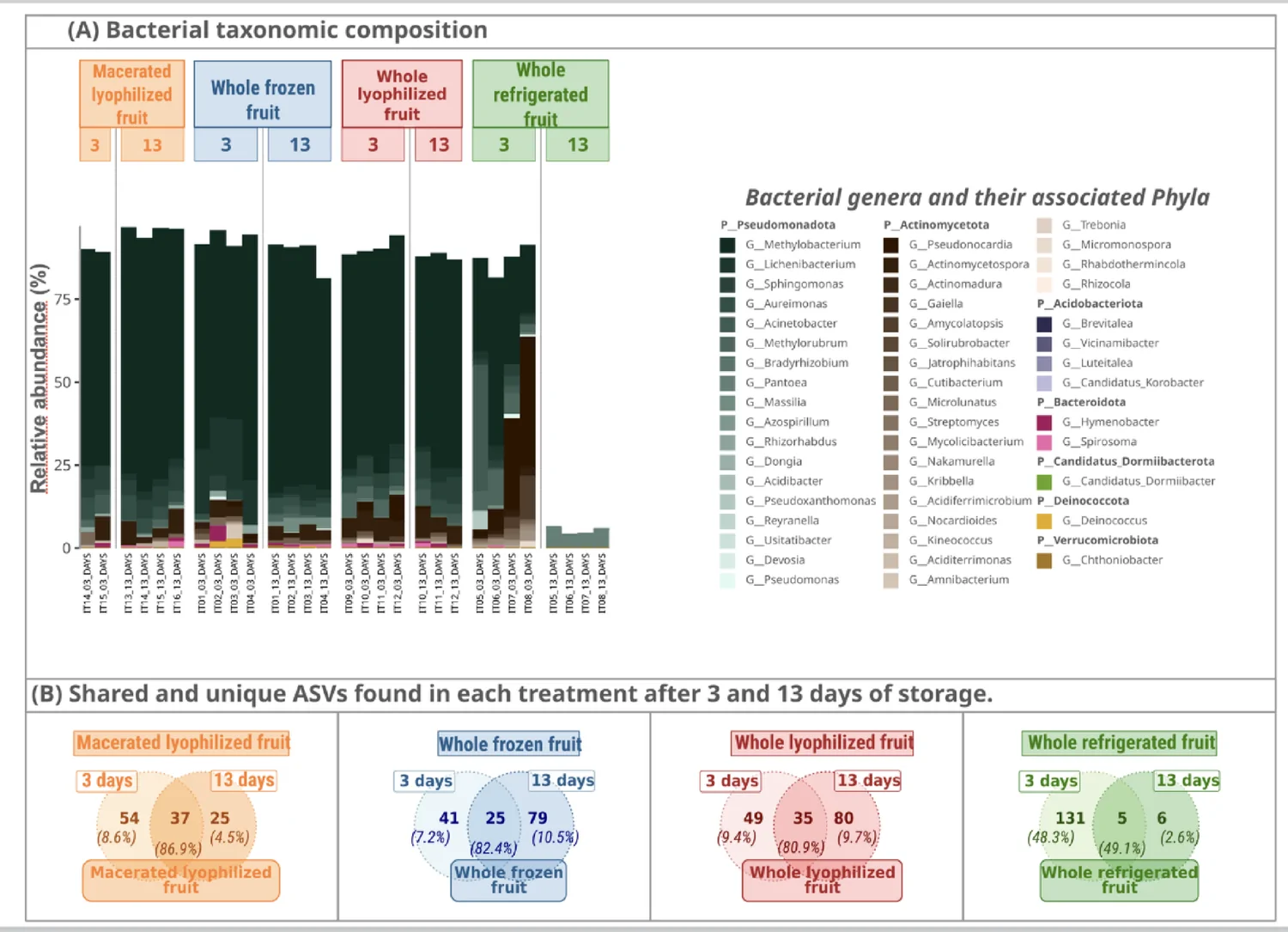
Taxonomic composition of bacteria at the genus level observed in all samples (A) and number of shared and unique Amplicon sequence variants (ASVs) detected in each treatment after 3 and 13 days (B). Only the 50 most abundant genera are shown. The greater the number of shared ASVs, the greater the preservation of the microbiota after 13 days of storage

The phylum Pseudomonadota dominated the bacterial community across all fruit treatments, with *Methylobacterium* and *Lichenibacterium* as the predominant genera in all treatments (Fig. 5A). Lyophilization treatments of both whole and macerated fruits maintained a stable taxonomic composition between days 3 and 13 (Fig. 5B), consistent with the high percentages of shared ASVs recorded (80.9% and 86.9%) and with the low PERMANOVA F-values (Fig. 2). The whole fruit frozen at − 80 °C likewise showed a stable composition (82.4% shared ASVs), although with a slight increase in Actinomycetota genera at day 13 and a minor accumulation of new ASVs (10.5%), suggesting modest community shifts during prolonged storage (Fig. 5). In contrast, refrigeration at 4 °C induced the most severe change: by day 13, the bacterial community that was diverse at day three underwent a drastic simplification dominated almost exclusively by *Pantoea* and *Pseudomonas.* This taxonomic collapse was reflected in the lowest percentage of shared ASVs among all treatments (49.1%; *n* = 5), with marked turnover of taxa detected between days 3 and 13, confirming that refrigeration at 4 °C is inadequate for preserving the integrity of the bacterial microbiome of coffee fruits beyond three days.

**Fig. 6:**
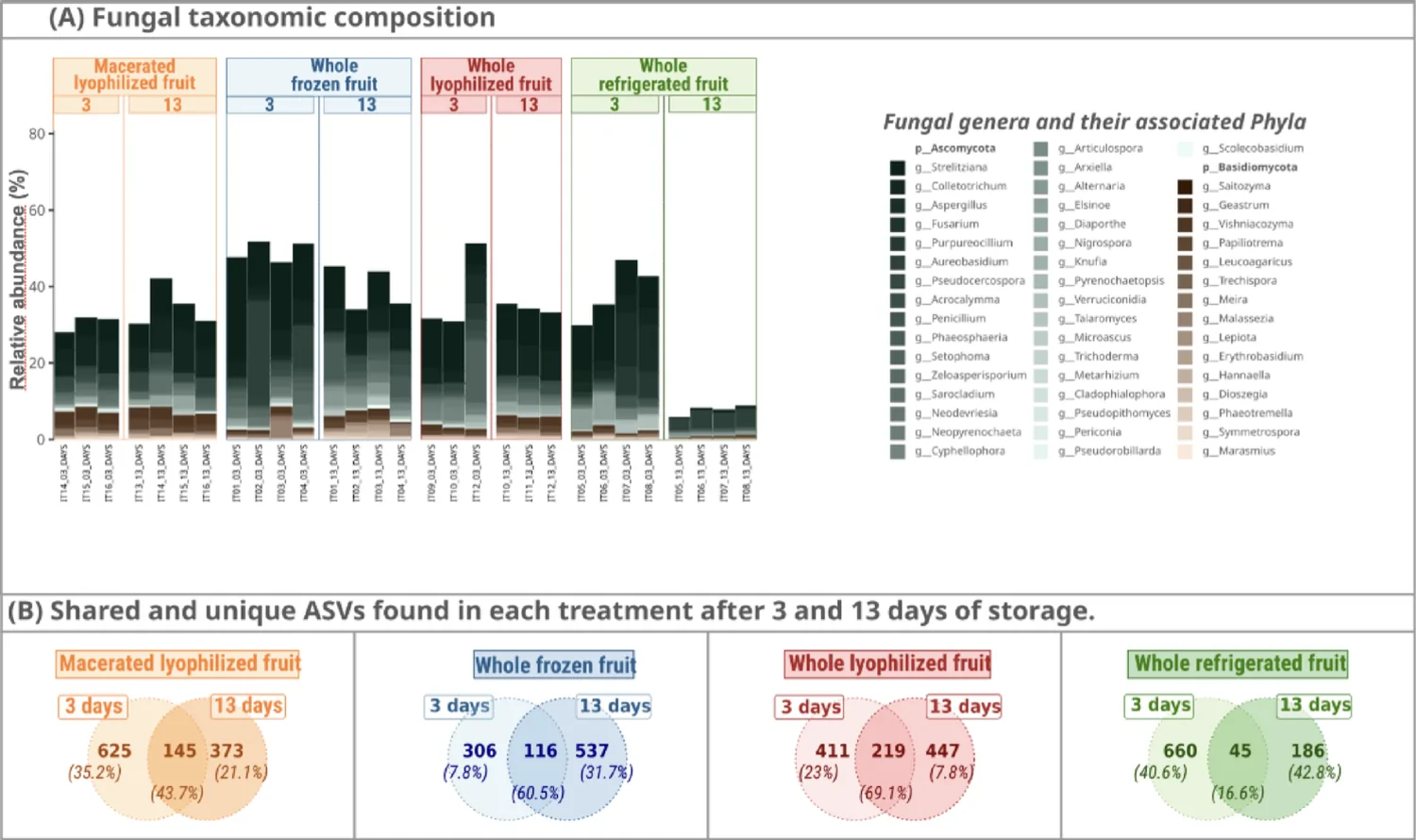
Taxonomic composition of fungi at the genus level observed in all samples (A) and number of shared and unique Amplicon sequence variants (ASVs) detected in each treatment after 3 and 13 days (B). Only the 50 most abundant genera are shown. The greater the number of shared ASVs, the greater the preservation of the microbiota after 13 days of storage

The phylum Ascomycota dominated the fungal community across all treatments, with *Strelitziana*, *Colletotrichum*, and *Aspergillus* as the predominant genera (Fig. 6A). Lyophilization treatments of both whole and macerated fruits maintained a stable taxonomic composition between storage times, with dominant fungal genera preserving their relative abundance across both time points (Fig. 6A). The whole fruit frozen at − 80 °C showed moderate stability, with slight fluctuations in *Fusarium* and *Purpureocillium* at day 13. However, refrigeration at 4 °C induced the most severe taxonomic simplification, with collapse of the native fungal diversity and dominance of a very limited number of taxa at day 13.

Regarding fungal ASV retention, the percentages of shared ASVs were consistently higher than those in bacterial communities across all treatments, suggesting a more stable core mycobiome over time. The lyophilized whole fruit (69.1%; *n* = 219) and whole fruit frozen at − 80 °C (60.5%; *n* = 116) showed higher shared fungal ASVs than other treatments (Fig. 6B). Whole fruit frozen at − 80 °C also exhibited a notable increase in unique fungal ASVs at day 13 (537; 31.7%). The lyophilized macerated fruit retained 43.7% of shared ASVs (*n* = 145), with greater taxon turnover possibly attributable to prior mechanical processing. In marked contrast, refrigeration at 4 °C presented the lowest retention of ASVs among all treatments (16.6%; *n* = 45), with a practically irreversible disruption of the mycobiome between days 3 and 13 (Fig. 6B).

Overall, lyophilization of whole fruits and freezing at − 80 °C were the most effective methods for preserving both the taxonomic structure and the identity of fungal taxa, while refrigeration at 4 °C proved inadequate for maintaining mycobiome integrity beyond three days of storage.

## Discussion

The preservation method was a primary determinant of microbiome stability in coffee fruits, with a clear ranking among treatments. However, ASV retention should be interpreted only as temporal retention between day 3 and day 13, rather than preservation relative to the original microbiome at harvest due to the absence of time-zero (T0) control. Consequently, our results compare the stability of physical preservation methods over time but cannot quantify absolute preservation of the initial microbial community.

Lyophilization minimized temporal shifts in beta diversity, followed by freezing at − 80 °C, whereas refrigeration at 4 °C induced the most pronounced alterations. These results reinforce that storage conditions critically influence DNA-based microbiome analyses (Lauber et al. 2010). The low PERMANOVA F-values observed under lyophilization indicate minimal structural disturbance, likely due to rapid dehydration halting microbial activity and host enzymatic processes. This high stability is consistent with findings in fecal and plant systems, where desiccation-based methods preserve microbial composition with limited variation (Camacho-Sanchez et al. 2013; Qiu et al. 2020), and suggests enhanced performance in metabolically active plant matrices.

Despite its effectiveness, lyophilization may introduce taxonomic biases. Previous studies reported increased Bacillota-to-Bacteroidota ratios, indicating preferential preservation of Gram-positive bacteria (Superdock et al. 2023). These biases are more evident in abundance-weighted metrics, emphasizing the importance of metric selection in comparative studies. Although previous studies have shown that chemical preservatives such as DESS can perform similarly to freezing in some biological matrices (Carvalhais et al. 2022), these preservation approaches were beyond the scope of the present study. Therefore, our results should be interpreted only as a comparison among the physical preservation strategies evaluated here and should not be extrapolated to chemical preservation methods.

Freezing at − 80 °C showed intermediate stability, maintaining overall community structure with moderate variation. Although widely considered a standard method, its performance in coffee fruits was lower than that reported for soil and fecal systems (Lauber et al. 2010; Shaw et al. 2016), indicating that plant-specific factors, including tissue enzymatic activity, introduce additional variability. This supports the need for matrix-specific preservation strategies, as also noted in plant microbiome studies (Qiu et al. 2020) and by evidence that storage conditions can alter microbial dominance patterns (Jenkins et al. 2018).

Refrigeration at 4 °C resulted in the most severe disruptions, with marked shifts in beta diversity and reduced ASV retention. Similar patterns have been described in fecal systems, where permissive temperatures rapidly favor opportunistic taxa (Shaw et al. 2016). In coffee fruits, these effects are likely amplified by tissue degradation and increased substrate availability, promoting the replacement of native communities. This observation reinforces concerns that refrigeration, although commonly used, may compromise microbiome integrity in plant studies (Qiu et al. 2020). Moreover, while low temperatures inhibit many metabolic processes, psychrotrophic microorganisms remain active and can dominate under these conditions (Senaratne et al., 2024; Yalew et al. 2024; Swanson et al., 2024; Yu et al. 2024).

Fungal communities were more temporally dynamic than bacterial communities, with consistently lower ASV retention, indicating higher sensitivity to post-sampling changes. Their similar response under freezing and refrigeration suggests that intrinsic biological traits, such as cell structure and stress tolerance, play a dominant role.

Alpha diversity patterns were consistent with beta diversity results. Refrigeration caused the greatest losses in richness and evenness, exceeding effects reported in fecal systems (Jenkins et al. 2018), whereas lyophilization maintained stable diversity over time, consistent with effective sample stabilization (Camacho-Sanchez et al. 2013). Freezing showed moderate variation, including increased richness, likely reflecting DNA release from damaged plant tissues rather than true ecological shifts (Lauber et al. 2010). Plant cells can sustain significant damage during cryopreservation, primarily due to extensive perforations caused by ice crystals that can expose the nuclear material (Pearce 2001; McAssey et al. 2023; Plitta-Michalak et al. 2025).

Taxonomic composition analyses supported these patterns. Core bacterial and fungal taxa were consistent with previous descriptions of the coffee microbiome (Vaughan et al. 2015; Veloso et al. 2020; Wiele et al. 2025; Rojas-Chacón et al. 2025). Lyophilization preserved dominant taxa and overall structure (Camacho-Sanchez et al. 2013), although potential phylum-level biases should be considered (Superdock et al. 2023). Freezing maintained core taxa with moderate fluctuations, particularly among low-abundance groups (Jenkins et al. 2018; Guo et al. 2016). In contrast, refrigeration led to a marked simplification of both communities, with dominance of opportunistic genera such as Pantoea and Pseudomonas, consistent with artificial restructuring reported under permissive conditions (Jenkins et al. 2018; Shaw et al. 2016), and compromising ecological interpretation (Rojas-Chacón et al. 2025; Veloso et al. 2020).

Overall, preservation method strongly determines microbiome integrity in coffee fruits. Lyophilization, particularly of whole fruits, provides the most reliable preservation of microbial diversity and composition, whereas refrigeration introduces substantial bias and should be avoided. Given the influence of the coffee microbiome on fermentation and beverage quality (Rojas-Chacón et al. 2025; Wiele et al. 2025; Veloso et al. 2020, 2023; Gomes et al. 2024), standardizing preservation protocols is essential to ensure reproducibility and ecological validity.

## Conclusions

This study demonstrates that preservation methods exert a significant and differential effect on the integrity of bacterial and fungal microbiomes in *Coffea arabica* fruits, with a clear ranking in preservation performance. Among the physical preservation methods evaluated, lyophilization of whole fruits was the most effective approach, consistently preserving alpha diversity, beta diversity, and microbial taxonomic composition. Lyophilization of macerated fruits and freezing at − 80 °C also showed high preservation capacity, whereas refrigeration at 4 °C proved inadequate for maintaining microbiome integrity beyond three days, leading to communities dominated by opportunistic taxa such as *Pantoea* sp. and *Pseudomonas* sp.

These findings have relevant methodological implications for coffee microbiome research. However, the absence of a time-zero (T0) control and chemical preservative comparisons, together with the restricted scope (single variety, location, and short storage period), warrants cautious interpretation of these findings. Therefore, ASV retention should be interpreted only as temporal retention between days 3 and 13, rather than preservation relative to the original microbiome at harvest. Although lyophilization appears to be a promising approach for field-based studies, particularly in remote regions where cold chain logistics are limited, further studies across different cultivars, environments, and storage durations are needed before establishing it as a standard preservation protocol.

## Data Availability

No datasets were generated or analysed during the current study.
